# In vitro assessment of nutraceutical compounds and novel nutraceutical formulations in a liver-steatosis-based model

**DOI:** 10.1186/s12944-018-0663-2

**Published:** 2018-02-05

**Authors:** Antonietta Stellavato, Anna Virginia Adriana Pirozzi, Francesca de Novellis, Ilaria Scognamiglio, Valentina Vassallo, Andrea Maria Giori, Mario De Rosa, Chiara Schiraldi

**Affiliations:** 1 Department of Experimental Medicine, Section of Biotechnology, Medical Histology and Molecular Biology, Università della Campania “Luigi Vanvitelli”, Naples, Italy; 2 R&D - IBSA Farmaceutici Italia, Lodi, Italy

**Keywords:** Steatosis in vitro model, PPARs expression, Oxidative stress, Nutraceutical compounds, HepG2, Normal liver cells

## Abstract

**Background:**

Steatosis is a chronic liver disease that depends on the accumulation of intracellular fatty acids. Currently, no drug treatment has been licensed for steatosis; thus, only nutritional guidelines are indicated to reduce its progression. The aim of this study is to combine different nutraceutical compounds in order to evaluate their synergistic effects on a steatosis in vitro model compared to their separate use. In particular, three different formulations based on silymarin, curcumin, vitamin E, docosahexaenoic acid (DHA), choline, and phosphatidylcholine were assayed.

**Methods:**

Human hepatocellular carcinoma cells (HepG2 cell line) were treated with a mixture of fatty acids in order to induce an in vitro model of steatosic cells, and then the amount of intracellular fat was evaluated by Oil Red O staining. The peroxisome proliferator-activated receptors α and γ (PPARα and γ) expression, closely correlated to lipid metabolism, was evaluated. The efficiency of these receptors was evaluated through the study of LPL mRNA expression, a marker involved in the lipolysis mechanism. Superoxide dismutase (SOD-2) and malondialdehydes (MDA) in lipid peroxidation were assayed as specific biomarkers of oxidative stress. In addition, experiments were performed using human liver cells stressed to obtain a steatosis model. In particular, the content of the intracellular fat was assayed using Oil Red O staining, the activation of PPARα and γ was evaluated through western blotting analyses, and the LPL mRNA expression level was analyzed through qRT-PCR.

**Results:**

All formulations proved effective on lipid content reduction of about 35%. The oxidative stress damage was reduced by all the substances separately and even more efficiently by the same in formulation (i.e. Formulation 1 and Formulation 3, which reduced the SOD-2 expression and induced the PPARs activation). Lipid peroxidation, was reduced about 2 fold by foormulation2 and up to 5 fold by the others compared to the cells pretreated with H_2_O_2_.Formulation 1, was more effective on PPARγ expression (2.5 fold increase) respect to the other compounds on FA treated hepathocytes. Beside, LPL was activated also by Formulation 3 and resulted in a 5 to 9 fold-increase respect to FA treated control.

**Conclusions:**

Our results proved that the formulations tested could be considered suitable support to face steatosis disease beside the mandatory dietetic regimen.

## Background

The accumulation of hepatic triglycerides results from an imbalance between their uptake, synthesis, and export, which leads to fatty acid oxidation. Prolonged hepatic steatosis can result in nonalcoholic steatohepatitis, fibrosis, and cirrhosis [1].

Several medical approaches have been proposed as steatosis therapies, but nowadays, there is not a specific treatment for this condition; only nutritional guidelines are used to prevent and/or treat this disease [2]. Recent studies reported the efficacy of natural compounds in steatosis treatment through the analyses of Peroxisome Proliferator-Activated Receptors (PPARs) used as biomarkers [3]. In fact, the literature reported that PPARs activation reduces fat accumulation, inflammation, and fibrosis in the in vitro model of steatosis [4]. Regarding hepatic metabolism, PPARα is strongly expressed in the liver, and its activation is related to fatty acid oxidation [5]. PPARγ operates as a lipid alert biomarker and plays a role in the pathogenesis of steatosis, increasing energy utilization [6]. For these reasons, PPARs agonists have been considered a potential pharmaceutical target for the treatment of steatosis [7]. In addition, fatty acids are activators of the PPARs and may increase the activity of lipoprotein lipase (LPL) [8]. The regulation of LPL through PPARs modulation may contribute to the beneficial fat reduction related to the crucial role of this enzyme in energy and lipoprotein metabolism in the liver [9]. In the framework of this research, our aim is to analyze the biological activity of three nutraceutical mixtures (Formulations 1, 2, 3) obtained from different combinations of six natural compounds that are well known for their effectiveness on steatosis: silymarin, curcumin, vitamin E, docosahexaenoic acid (DHA), choline, and phosphatidylcholine. Only Formulation 1 is composed of all these molecules; Formulations 2 and 3 do not contain choline and phosphatidylcholine, respectively. Silymarin, or milk thistle extract, is an herbal remedy of the European tradition, used (also as a drug) to treat and prevent injuries and pathologies of the liver [10]. Curcumin is a well-known active plant ingredient that has shown a promising hepatoprotective effect in animal models [11]. Vitamin E [12] and omega-3 fatty acid are already used as nutraceuticals for human treatments [13]. Studies about vitamin E treatment showed discordant results. In vitro vitamin E proved effective in reducing oxidative stress in liver cells [14]. In vivo studies on pediatric patients showed that the compound improved steatosis activity scores, but the effects apparently were not significantly different with respect to the placebo. However, few data are now available on the effect of antioxidants associated with vitamin E in children affected by steatosis in which there is a significant improvement of the liver function and glucose metabolism [15]. Because of the better performance demonstrated by combinations/mixtures of different compounds compared to the single-molecules-based products, new formulations are going to be evaluated and eventually commercialized. Pediatric steatotic patients, after 12 months of treatment with dietary supplements, exhibited observable improvement in insulin resistance, hepatic enzyme levels, and liver histology, without an increase in body weight [16]. Choline, also in the form of phosphatidylcholine, is a food ingredient with an approved health claim issued by the European Food Safety Agency (EFSA) for “maintaining healthy liver functioning” [17]. For this reason, in the present research work, besides the evaluation of single compounds, we assayed three formulations in order to evaluate the potential synergistic effect of novel nutraceutical combinations on an in vitro model of liver steatosis.

## Methods

### Reagents

All used nutraceutical compounds are commercially available food ingredients: sylimarin and curcumin (turmeric extract complexed with soy phosphatidylcholine - Meriva®) were supplied by Indena (Milan, Italy), vitamin E (D-Alpha Tocopherol 1000 IU/g) by Van Eeghen (Amsterdam, The Netherland), choline bitartrate (VitaCholine) by Balchem (Marano Ticino, Italy), phosphatidylcholine (PHOSAL® 40 IP) by Lipoid (Steinhausen, Switzerland) and DHA 70% (Omegavie DHA 70 EE Qualitysilver Ice) by Polaris (Pleuven, France). The experiments were performed using nutraceutical ingredients tested as powder solubilized in Dimethyl sulfoxide (pure DMSO) solution and then diluted to use them as single and/or as mixtures in serum-free medium at a final concentration of 0.5% *v*/v DMSO, that proved not toxic to the cells.

### Cell culture

HepG2 cell line was provided by ATCC Cell Biology Collection and cultured in Dulbecco’s modified Eagle’s medium (DMEM) supplemented with 10% *v*/v FBS, 100 U/mL penicillin, 100 μg/mL streptomycin and 100 μg/mL antifungal. Normal human liver cells were provided by LONZA (HUCPI, Lot.HUM4056B), Euroclone and cultured in LONZA completed medium. All cell culture materials were purchased from Gibco (Invitrogen, Milan, Italy). The cells were grown on tissue culture plates (BD Bioscience-Falcon, San Jose, USA), using an incubator with a humidified atmosphere 95% *v*/v air to 5% CO2 v/v at 37 °C.

### In vitro model of steatosis

In vitro steatosis, as reported in the study of Gaens and collaborators (2012) [18] was induced by incubating HepG2 cells or human normal liver cells (1.0 × 10^5^ cells/well seeded in a standard 24-well culture plate) with 6 mM of a mixture of linoleic acid (a polyunsaturated 18:2,ω-6 fatty acid) and oleic (monounsaturated 18:1, ω-9 fatty acid) ratio 1:1 *v*/v (L9655-Sigma Aldrich, Milan, Italy) (FAs) for 24 h [19]. After a PBS wash, cells were exposed for an additional 24 h either to the single natural compounds or to the new potential nutraceutical formulations.

### In vitro model of oxidative stress

HepG2 cells (1.0 × 10^5^ cells/well in a standard 24-well culture plate) were pre-treated for 30 min with 50 μM H_2_O_2_ and then incubated with the nutraceutical ingredients for 24 h.

### Staining

Intracellular fatty acids amount was evaluated by Oil Red O staining (0.5% v/v) (Sigma Aldrich, Milan Italy). Cells pictures were captured by an optic microscope (Niko Eclipse TS 100) and stained lipid droplets were then extracted with isopropanol (60% v/v) and quantified by measuring the absorbance at 510 nm [20]. For Oil Red O staining three concentrations (0.001–0.005-0.05 mg/mL) were screened for the single substances, while for all other experiments, single molecules were tested at 0.001 mg/mL and formulations at 0.005 mg/mL, as sum up of the concentration of all the components.

### Cell proliferation and viability (MTT test)

Cytotoxicity was assessed in HepG2 cells (1.0 × 10^5^ cells/well in a standard 24-well culture plate) by measuring the reduction of the tetrazolium dye 3-(4, 5-dimethylthiazol-2-yl)-5-(3 carboxymethoxyphenyl)-2-(4- sulfophenyl)-2H–tetrazolium (MTT) [21]. The cytotoxic effect is evaluated by the percentage of living cells present in the sample, in relation to the cells treated only with the solutions. The optical densities of the obtained solutions were measured at 570 nm using a Beckman DU 640 spectrometer (Beckman, Milan, Italy).

### RNA isolation and quantitative RT-PCR

To better assess the oxidative stress, we analyzed the expression levels of SOD-2 using the specific primer pairs: forward primer *5’-CTGGACAAACCTCAGCCCTA-3′* and reverse primer *5′-TGA TGGCTTCCAGCAACTC-3′*, through quantitative RT-PCR (qRT-PCR), (BioRad iQ5 Multicolor Real Time PCR Detection System). HepG2 cells (1.0 × 10^5^) after 24 h from seeding, were treated with hydrogen peroxide (H_2_O_2_, 50 μM) for 30 min and next with all nutraceutical compounds (0.001 mg/ml). After 24 h of treatment, total RNA was isolated using Trizol reagent (Invitrogen Milan, Italy) following the manufacturer’s instructions. RNA concentrations were measured spectrophotometrically using a Nanodrop Instrument (Celbio, Milan Italy). One microgram of total RNA was reversely transcribed into cDNA with Reverse Transcription System Kit (Promega, Milan, Italy) according to the manufacturer’s instructions. Quantitative real-time PCR was performed using IQ ™ SYBR® Green Supermix (Bio-Rad Laboratories, Milan, Italy). Besides**,** LPL was analyzed as biomarker of lipid metabolism. In the specific, 1.0 × 10^5^ human hepatocytes were seeded for mRNA evaluations using the specific primer pairs: forward primer *5’-*AATGAGGTGGCAAGTGTCCT*-3′* and reverse primer *5’-*CTCCAGAGTCTGACCGCCT *-3.* The gene expression of SOD-2 and LPL was normalized respect to the expression level of the housekeeping gene using the specific GAPDH primer pairs: forward primer *5’*-*TGCACCACCAACTGCTTAGC-3′* and reverse primer *5′- GGCATGGACTGTGGTCATGAG -3′* [19–22].

### Western blotting analyses for SOD-2, PPARα and γ

SOD-2, PPARα and γ expression markers were assayed through western blotting analyses. The proteins have been extracted using Radio-Immunoprecipitation Assay (RIPA) buffer R0278 (Sigma Aldrich, Milan, Italy) and the concentrations were determined using the Bio-Rad protein assay reagent (Bio-Rad Laboratories, Milan Italy). Equal amounts of protein (30 μg) were loaded on a SDS-PAGE and transferred to a nitrocellulose membrane [19–22]. The filters were incubated with a monoclonal antibody against SOD-2 (A-2, sc-133,134) diluted 1:250 *v*/v, polyclonal antibody against PPARα (H-98, sc-9000) diluted 1:200 v/v, monoclonal antibody against PPARγ (E-8, sc-7273) diluted 1:250 v/v, polyclonal antibody against Actin (I-19, sc-1616) diluted 1:500 v/v, at room temperature for 2 h. All reagents were from Santa Cruz Biotechnology (Santa Cruz, CA, USA). Membranes were washed three times for 10 min and incubated with a 1:10,000 v/v dilution of horseradish peroxidase-conjugated anti-mouse, anti-rabbit and anti-goat antibodies for 1 h respectively. Blots were developed using ECL system (Amersham Biosciences, UK) according to the manufacturer’s protocols.

### Lipid peroxidation assay (TBARs)

Since oxidative stress is closely associated to liver steatosis development, TBARS assay was also used to evaluate lipid peroxidation. Hydrogen peroxide (H_2_O_2_) 1 M solution, thiobarbituric acid (TBA), trichloroacetic acid (TCA), hydrochloric acid (HCl), paraformaldehyde and isopropanol reagents were from Sigma Aldrich (Milan Italy). The protein concentrations were determined using the Bio-Rad protein assay reagent (Bio-Rad Laboratories, Milan Italy). Lipid peroxidation was evaluated using the quantification of Thiobarbituric Acid Reactive Substances (TBARS) as previously reported [23]. All data showed as reduction percentage respect to H_2_O_2_ are the means ± standard deviation (SD) of 3 experiments and each experimental point is the average of triplicate measurements.

### Statistical analysis

All of the experimental results were expressed as mean ± standard deviation (SD) of at least three independent determinations for each experiment. Differences between all treatments were tested for statistical significance by Student *t* test, *p* values lower or equal to 0.05 were considered significant.

## Results

### Quali-quantitative analyses of oil red O staining to assess steatosis level

**Fig. 1:**
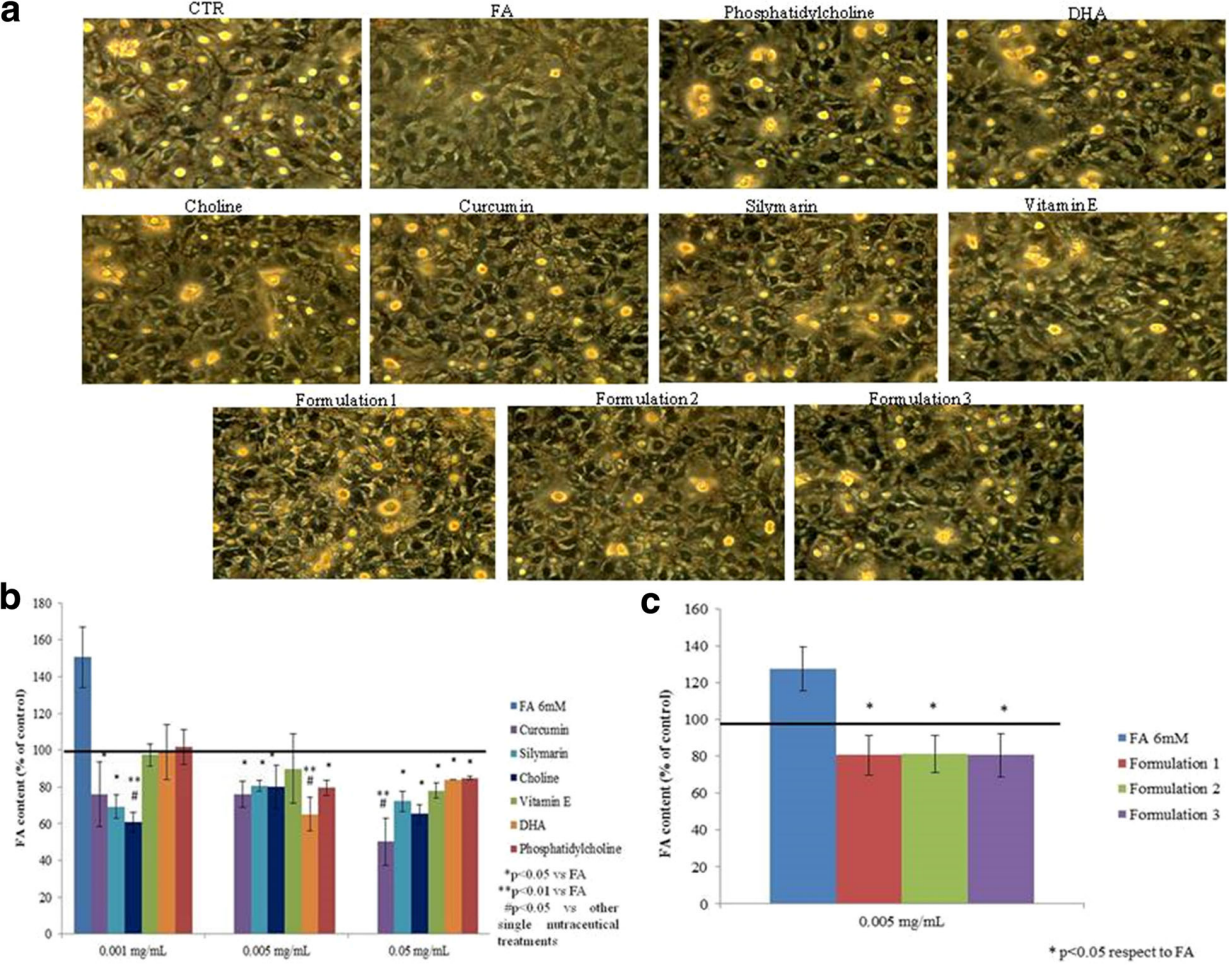
In vitro steatosis, Oil Red O staining colorimetric assay. **a** Oil Red O staining pictures for HepG2 cells in presence of fatty acids (FA) 6 mM, different nutraceutical compounds (0.001 mg/mL) and their formulations (0.005 mg/mL). **b** Spectophotometric quantification and evaluation of lipid amount percentage with the different nutraceutical compounds (concentration range 0.001–0.05 mg/mL) respect to the control of steatotic cells (FA 6 mM). Curcumin, sylimarin vs CTR * that means *p* < 0.05 at 0.001 mg/ml. Choline vs CTR ** that means *p* < 0.01 at 0.001 mg/ml, and vs vitamin E ^#^, DHA ^#^ and phosphatidylcholine ^#^ that means *p* < 0.05 at 0.001 mg/ml. Curcumin, sylimarin, choline and phosphatidylcholine vs CTR * that means *p* < 0.05 at 0.005 mg/ml. DHA vs CTR ** that means *p* < 0.01 at 0.005 mg/ml. DHA vs sylimarin ^#^ that means *p* < 0.05 at 0.005 mg/ml. Sylimarin, choline, vitamin E, DHA and phosphatidylcholine vs CTR * that means *p* < 0.05 at 0.05 mg/ml. Curcumin vs CTR ** that means *p* < 0.01 at 0.05 mg/ml. Curcumin vs DHA ^#^ and phosphatidylcholine ^#^ that means *p* < 0.05 at 0.05 mg/ml. **c** The graph reported Oil Red O staining colorimetric assay relative to lipid droplets accumulation in HepG2 cells treated with different nutraceutical formulations (0.005 mg/mL). All values were expressed in the form of mean ± SD (*n* = 3). **b** and **c** are relative to two groups of different experiments in which intracellular fat difference in the averaged data proved not to be significant using t-student test *p* > 0.05

**Fig. 2:**
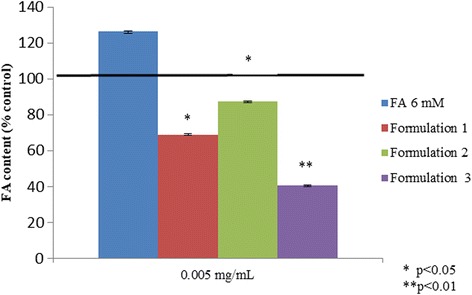
In vitro steatosis model, Colorimetric assay (Spectophotometric quantification) of Oil Red O staining for human normal hepatocytes in presence of fatty acids (FA) 6 mM and nutraceutical formulations (0.005 mg/mL) normalized on control

Oil Red O staining showed that treatment with the oleic and linoleic acid mixture induced the accumulation of lipid droplets in almost all HepG2 cells, as shown in the micrographs presented in Fig. 1a. Nutraceutical compounds were already active in reducing lipid accumulation at the 0.001 mg/mL concentration (Fig. 1b). In particular, the reduction fold, with respect to the FA treatment in the presence of curcumin, was 1.6 fold (*p* < 0.05). With silymarin, it was 1.7 fold (*p* < 0.05), and when using choline, it reached 2.0 fold (*p* < 0.01). Additionally, the assays showed that lipid droplets are even lower when the cells are treated with all of the formulations developed (1, 2, 3) at 0.005 mg/mL, compared to the control (Fig. 1c). The differences were statistically significant in all pairs of data tested. Above all, in reducing the FA content, sylimarin and curcumin showed significant differences in comparison with the other single substances, tested at the lowest concentration. Also, the experiments performed on normal human liver cells showed that treatment with an oleic and linoleic acid mixture induced the accumulation of lipid droplets, while the formulations reduced the content of the intracellular fat. In particular, all three formulations are significantly effective on steatosis (*p* < 0.05), but Formulation 3 showed more effectiveness on lipid reduction (*p* < 0.01) compared to the others, as reported in Fig. 2.

### Liver cells exposed to oxidative stress in the presence of nutraceuticals and their formulations: Cell viability assay

**Fig. 3:**
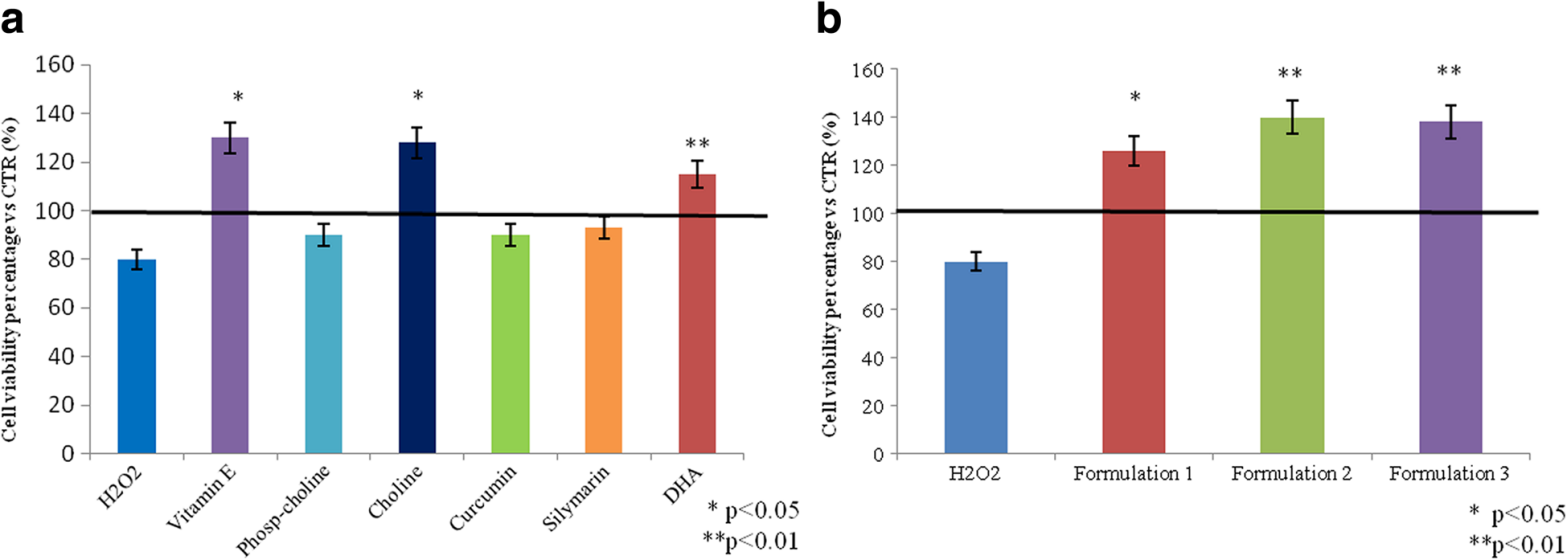
MTT assay for single nutraceutical compounds (**a**) and formulations (**b**). The figure shows the cell viability percentage of HepG2 cells after 30 min of H_2_O_2_ (50 μM) treatment and after nutraceutical compounds incubation. **b** and **c** are relative to two groups of different experiments in wich intracellular fat difference in the averaged data proved not to be significant using t-student test *p* > 0.05

Cell viability was evaluated using the MTT test after 24 h of exposure to nutraceutical molecules at different concentrations (Fig. 3a,b). The compounds were not cytotoxic for all treatments tested. In particular, vitamin E (*p* < 0.01), choline (*p* < 0.01), and DHA (*p* < 0.05) were the only single compounds that not only were not cytotoxic at the concentration tested, but also were involved in cell proliferation. All of our formulations increased cell viability and proliferation.

### Oxidative stress: SOD- 2 gene and protein expression

**Fig. 4:**
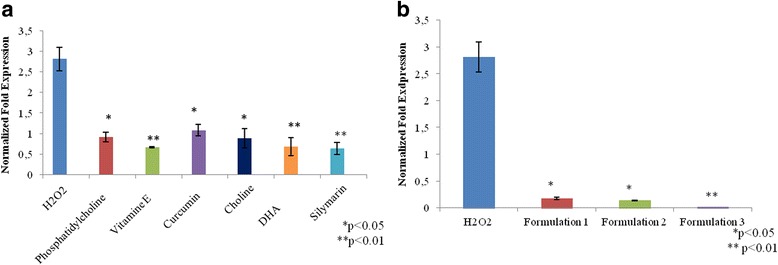
**a**, **b** Oxidative stress. The figure shows the anti-oxidant effect of nutraceutical compounds in HepG2 cells, after 30 min of H_2_O_2_ (50 μM) treatment through the  gene expression analyses of SOD-2

**Fig. 5:**
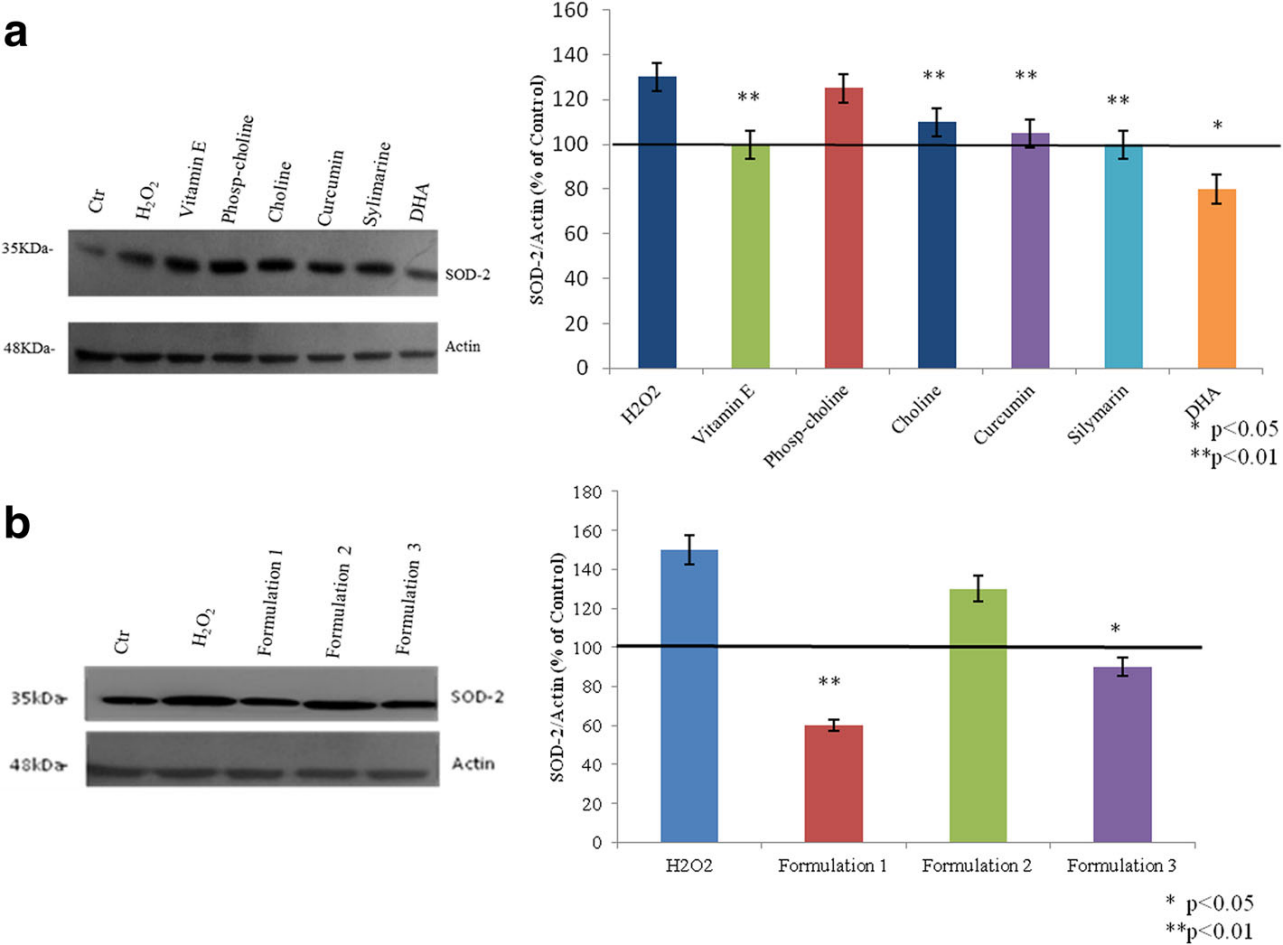
**a**, **b** Western blot relative to SOD-2 as marker of oxidative stress. Actin is used to normalize the results. All values were expressed in the form of mean ± SD (*n* = 3)

In order to evaluate the antioxidant effect of nutraceutical molecules, SOD-2, a well-established key marker, was analyzed. As shown in Fig. 4a, all of the molecules were investigated separately, and their formulations reduced the SOD-2 mRNA expression with respect to the positive control. In particular, vitamin E, DHA, and silymarin exert an efficient antioxidant role, significantly reducing SOD-2 with respect to the control (*p* < 0.01). All of the formulations proved even more active than the single molecules; in addition, Formulation 3 was the most effective among the three different formulations tested (Fig. 4b) (*p* < 0.01). Furthermore, western blotting analyses (Fig. 5a) showed a significant SOD-2 reduction in the presence of DHA alone (*p* < 0.01); similarly, Formulation 1 (*p* < 0.01) and Formulation 3 (*p* < 0.05) were effective, as reported in Fig. 5b.

### Evaluation of PPARα and PPARγ expression

**Fig. 6:**
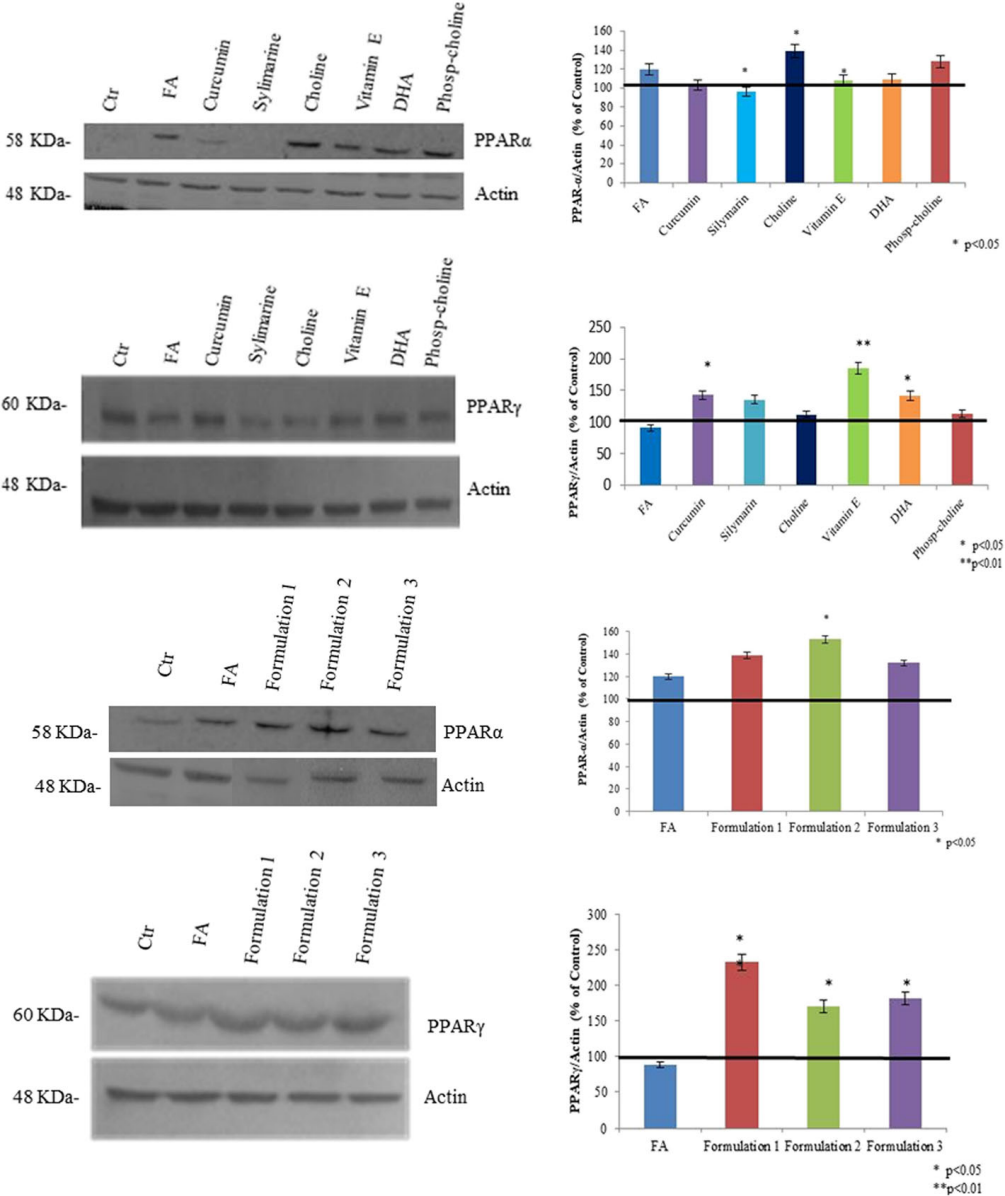
PPARα and PPARγ protein expression level on HepG2 cells determined by Western blotting. Actin is used to normalize the results

**Fig. 7:**
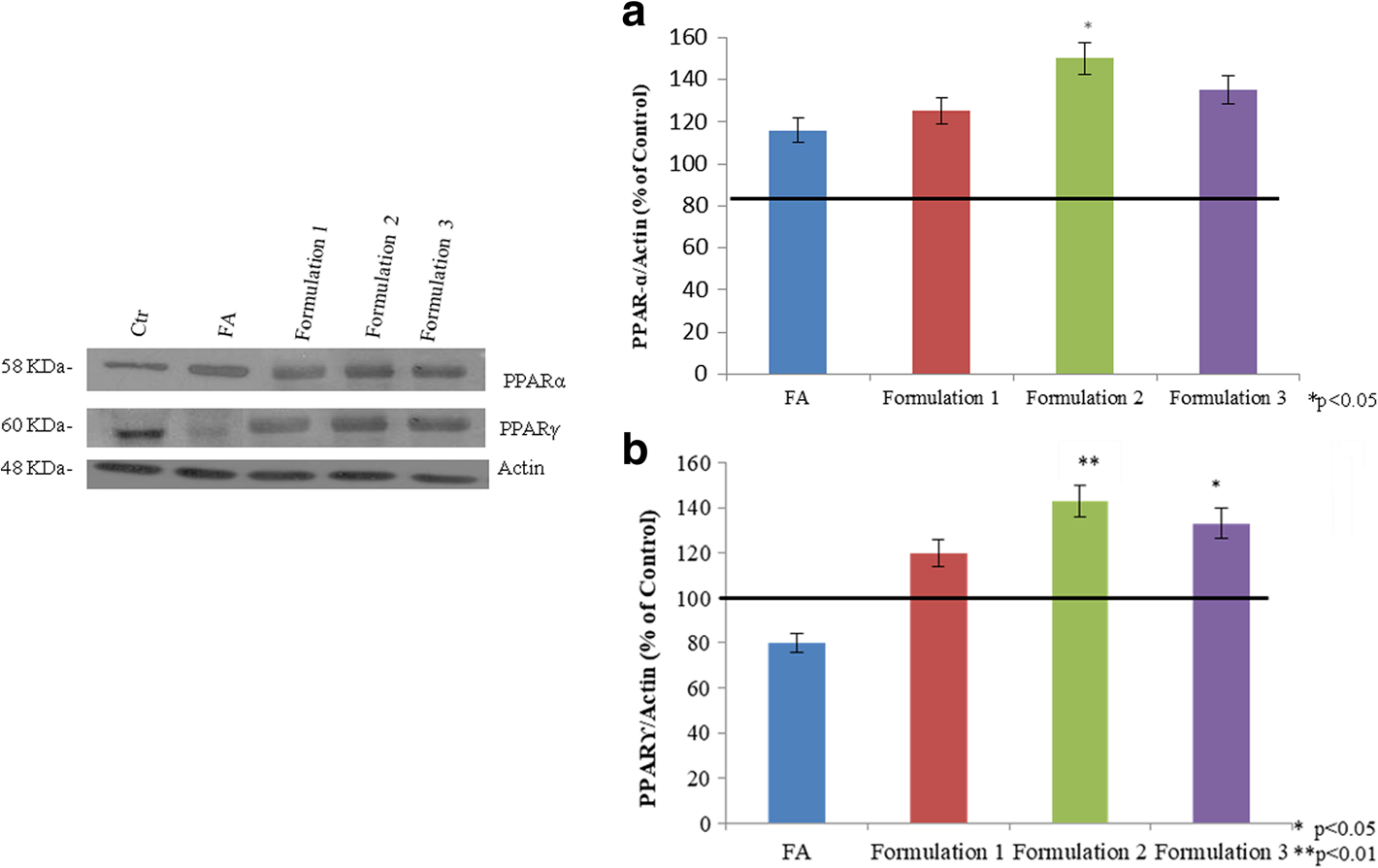
**a**, **b** PPARα and γ protein expression level determined by western blotting in human normal liver cells, exposed to FA to induce steatosis and eventually treated with nutraceuticals. Actin is used to normalize the results

PPARα and γ were detected by western blot analyses. The results showed that PPARα was increased in the presence of fatty acids, maybe as a cellular physiological response. This increment is more evident when HepG2 cells were treated by nutraceutical compounds, in particular, in the presence of choline (*p* < 0.05), phophatidylcholine, and Formulation 2 (*p* < 0.05) (Fig. 6). PPARγ (Fig. 6) was significantly decreased when the HepG2 were treated with FA, with respect to the control (*p* < 0.01). However, its expression was upregulated by silymarin (*p* < 0.05), vitamin E (*p* < 0.01), curcumin (*p* < 0.05), and DHA (*p* < 0.05), reverting the steatotic condition to the basal cells state (Fig. 6). Finally, all of the formulations increased the expression of PPARγ even more than the single substances. In our in vitro model, Formulation 1, which included all of the natural compounds tested, was more effective on PPARγ activation, up to 2.5 fold (*p* < 0.01), compared to the others (Fig. 6). The formulations were also tested on normal liver cells treated with FA; the experiments showed that all of the formulations are able to increase PPARα expression. In particular, there was a significant increase in cells treated with Formulation 2 (*p* < 0.05), compared to the hepatocytes treated with FA (Fig. 7a). The PPARγ expression was decreased in the cells treated with FA, but in the presence of nutraceutical formulations, it was upregulated, particularly with Formulation 2 (*p* < 0.01) and Formulation 3 (*p* < 0.05) (Fig. 7b).

### LPL expression related to lipid metabolism

**Fig. 8:**
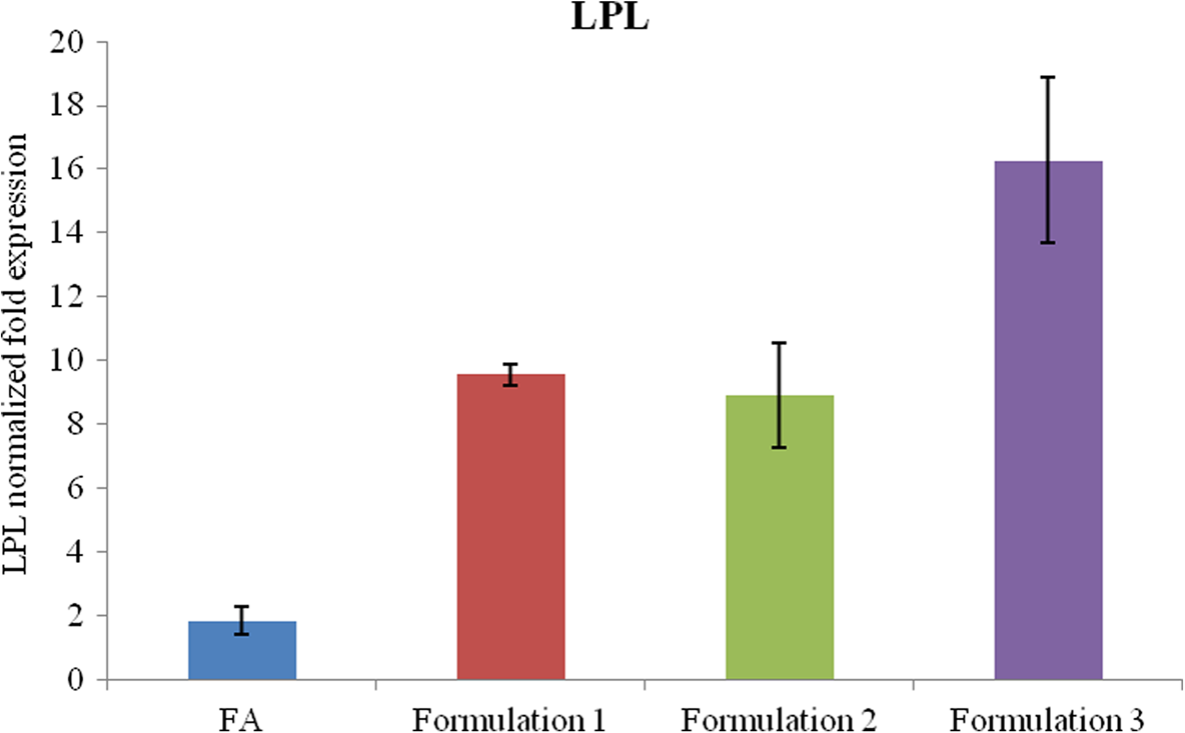
LPL gene expression on human normal liver cells in presence of nutraceutical formulations

In the Fig. 8 is reported the expression levels of LPL in normal human hepatocytes. The cells treated with fatty acids induced LPL up-regulation 2-foldrespect to control. The LPL mRNA expression showed a 9-fold increase when the cells are treated with Formulation 3 and about 5 fold increase for formulation 1 and 2, when compared to the FA treatment.

### Lipid peroxidation analyses

**Fig. 9:**
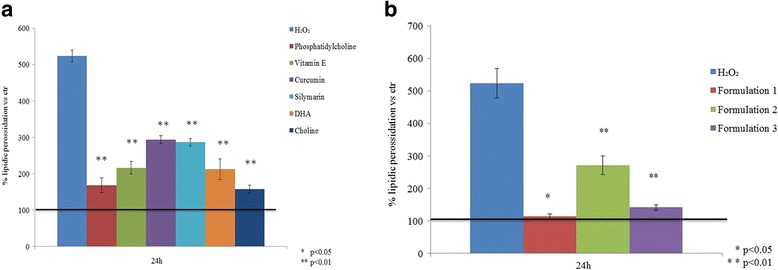
**a** Effect of single nutraceutical compounds (0.001 mg/mL) on lipid peroxidation after exposure to oxidative stress induced by 50 μM H_2_O_2_ in vitro on HepG2 cells. **b** Lipid peroxidation in presence of nutraceutical formulations. Data are the means ± SD (*n* = 3) and were significantly different: * *p* < 0.05 and ** *p* < 0.01 respect to H_2_O_2_ pre-treatment

A lipid peroxidation assay was used to investigate the effect of natural compounds on H_2_O_2_ oxidative stress; as shown in Fig. 9, a significant decrease of TBARs was observed in the treated cells. A superior reduction of lipid peroxidation was found with phosphatidylcholine, choline (Fig. 9a), and formulation treatments (Fig. 9b). With Formulation 1, there was approximately a 4-fold reduction (*p* < 0.05) compared to the cells pretreated with H_2_O_2_; when using Formulation 3, TBARs are reduced to the untreated-cells level, thereby favouring the reestablishment of the physiological (oxidation) state (*p* < 0.01) (Fig. 9b).

## Discussion

In our experimental study, we evaluated the potential antisteatotic and antioxidant activities of different nutraceutical compounds, which have already been suggested for their potential beneficial effects on liver metabolism. As reported in the literature, natural compounds are able to bind and activate PPARs [6]. Actually, the commercial demand for dietary supplements has been increased in the treatment of metabolic disease (e.g., steatosis), thanks to their potential positive influence on human health. In recent scientific literature, vitamin E [24], curcumin [25], silymarin [26–29], DHA [30], choline, and phosphatidylcholine were reported to have a positive effect on cellular physiology [31, 32]. Our results confirmed that the formulations modulated better than single nutraceutical compounds the analysed pathological mechanisms of steatosis. In particular fatty acids accumulation, PPAR-α and -γ receptors expression [6], and oxidative stress [33] were investigated. Several studies reported that PPAR-α and -γ receptors are the key molecules in the regulation of lipid metabolism with different functions. In particular, PPAR-α promotes fat mobilization, while PPAR-γ is involved in fat storage; its overexpression may prevent the hepatic steatosis in amurine hepatic steatosis model of [34]. In our in vitro study, the PPARα and γ are modulated by all formulations in both cellular models, corroborating the scientific literature results. In addition, it is known that PPARs signaling pathway modulate LPL activity, that is responsible of the fatty acids hydrolysis (in particular triglycerides). In this respect, we investigated the possible mechanism that follows PPARs activation, and we selected LPL as a marker of lipid metabolism [35]. In particular, Oil Red O staining quantification concerning single compounds showed that only curcumin, silymarin (*p* < 0.05), and choline (*p* < 0.01), at a 0.001 mg/mL concentration, significantly reduced the lipid accumulation by about 50–70% compared to the FA positive control into HepG2 cells. All three nutraceutical formulations showed a significant (*p* < 0.05) reduction in the lipid amount. The results obtained for nutraceutical formulations have also been confirmed in normal hepatocytes; in fact, all of them significantly reduced the intracellular lipid droplets (*p* < 0.05) with respect to the FA treatment. In particular, Formulation 3 was the most effective on lipid reduction (*p* < 0.01). Moreover, our results showed that LPL is upregulated in the presence of Formulation 3. Because the excessive FA amount causes peroxidation in the liver, we also evaluated the protective effects of these formulations on oxidative stress (ROS), quantifying the SOD-2 expression at a transcriptional and protein level in a model based on HepG2 cells. Our data demonstrated that the SOD-2 gene expression is significantly reduced in the presence of all compounds, compared to a positive control (H_2_O_2_); in particular, vitamin E, silymarin, and DHA showed the best results. The results on vitamin E were expected and are in agreement with the initial in vitro studies of Barja and collaborators [36], which described its role as a natural antioxidant. More interesting, DHA, an essential fatty acid of the ω-3 series, was found to have a relevant activity on fat accumulation in liver cells, reducing the inflammation in pediatric patients through an NF-kB pathway modulation [37]. Thus, different nutraceutical compounds exert a role in controlling human dyslipidaemia. Experimental results corroborated that functional foods ingredients (e.g. resveratrol, DHA, curcumin, zinc) may enhance lipid metabolism ameliorating, among the different lipid dysfunctions comprising the hypercholesterolemia. In this respect the authors discussed that nutraceutical compounds effectively reduced liver fatty acid accumulation both counteracting the activity of the enzymes responsible of lipid synthesis and intensifying the activity of degradation enzymes such as LPL [38]. Nowadays, it is possible to find commercialized food supplements based on DHA-choline to modulate lipid metabolism and reduce their accumulation. A recent review presented by Gualiang and collaborators presented steatosis as the “most universal and severe chronic liver disease worldwide” in the future, and in relation to the pathogenetic complex mechanisms, the authors suggested the contemporary use of micronutrients, such as vitamin E and sylimarin, as a preventive and therapeutic strategy [24]. Our results prompted similar conclusions, showing that the nutraceutical activity of vitamin E and silymarin, can be implemented by DHA and choline for their specific functions, and curcumin, which shows bioactivity against ROS. In light of the new results, it was possible to correlate the PPAR-α expression to LPL activity. In normal hepatocytes, PPAR-α is activated by the formulations, and this up regulation is directly related to LPL expression, suggesting its key role in lipid metabolism. In this respect, the study of the biological modulation of PPARs may help to establish, the efficacy of nutraceuticals to assess their therapeutic potential in metabolic liver disease. In the framework of this research, the formulations tested proved all active, however few differences may be pointed out. In fact, formulation 3, which contains all of the nutraceutical components except for phosphatidylcholine, is able to advantageously modulate all of the biomarkers involved in the development of steatosis. Therefore, among all, the latter could represent a new potential product for the treatment of this disease.

## Conclusions

The results presented in this in vitro experimental work suggest that all of the natural compounds and their formulations were able to counteract fat accumulation and liver oxidative stress damage. Between the single natural substances and their combinations we observed a synergistic effect in Formulations 1 and 3. In particular, studying in depth the molecular mechanism activated from PPARs toward LPL overexpression, we found that, only Formulation 3 activates the lipolysis pathway at various levels. The interesting results obtained, are based on an in vitro model, therefore they only represents a potential systematic pre-clinical approach suitable to compare natural substances on their prospective impact on fat over-accumulation in the liver. This system has the limitation of a lack of external factors that could influence hepatocyte behaviour in vivo, as reported in Gomez et al. (2006) [39]. Thus, a final efficient comparison should be based on an animal model to better unravel the bioactivity of the nutraceutical compounds and their formulations in mammalians.

## Abbreviations

DHA: Docosahexaenoic acid
DMEM: Dulbecco’s modified Eagle’s medium
DMSO: Dimethyl sulfoxide
EFSA: European Food Safety Agency
FA: Fatty acids
H_2_O_2_: Hydrogen peroxide
HepG2: Human hepatocellular carcinoma
LPL: Lipoprotein lipase
PPARs: Peroxisome proliferator-activated receptors
RIPA: Radio-Immunoprecipitation Assay buffer
TBA: Thiobarbituric acid
TBARS: Thiobarbituric Acid Reactive Substances
TCA: Thydrochloric acid
