# Correction to: In vitro assessment of nutraceutical compounds and novel nutraceutical formulations in a liver-steatosis-based model

**DOI:** 10.1186/s12944-022-01646-6

**Published:** 2022-04-06

**Authors:** Antonietta Stellavato, Anna Virginia Adriana Pirozzi, Francesca de Novellis, Ilaria Scognamiglio, Valentina Vassallo, Andrea Maria Giori, Mario De Rosa, Chiara Schiraldi

**Affiliations:** 1 Department of Experimental Medicine, Section of Biotechnology, Medical Histology and Molecular Biology, Università della Campania “Luigi Vanvitelli”, Naples, Italy; 2 R&D - IBSA Farmaceutici Italia, Lodi, Italy

**Correction to: Lipids Health Dis 17, 24 (2018)**

**https://doi.org/10.1186/s12944-018-0663-2**

Following the publication of the original article [1], the authors noticed an inaccuracy related to western blotting actin bands in Figs. 5 and 6.

In this respect, the authors apologize for uploading the western blotting (actins bands) that could be misleading. The actins obtained for the filters incubated for SOD-2 antibody were incorrectly duplicated for filters incubated with PPAR-γ. Presented here are the correct Figs. 5 and 6 with the correct actins references.

**Fig. 5:**
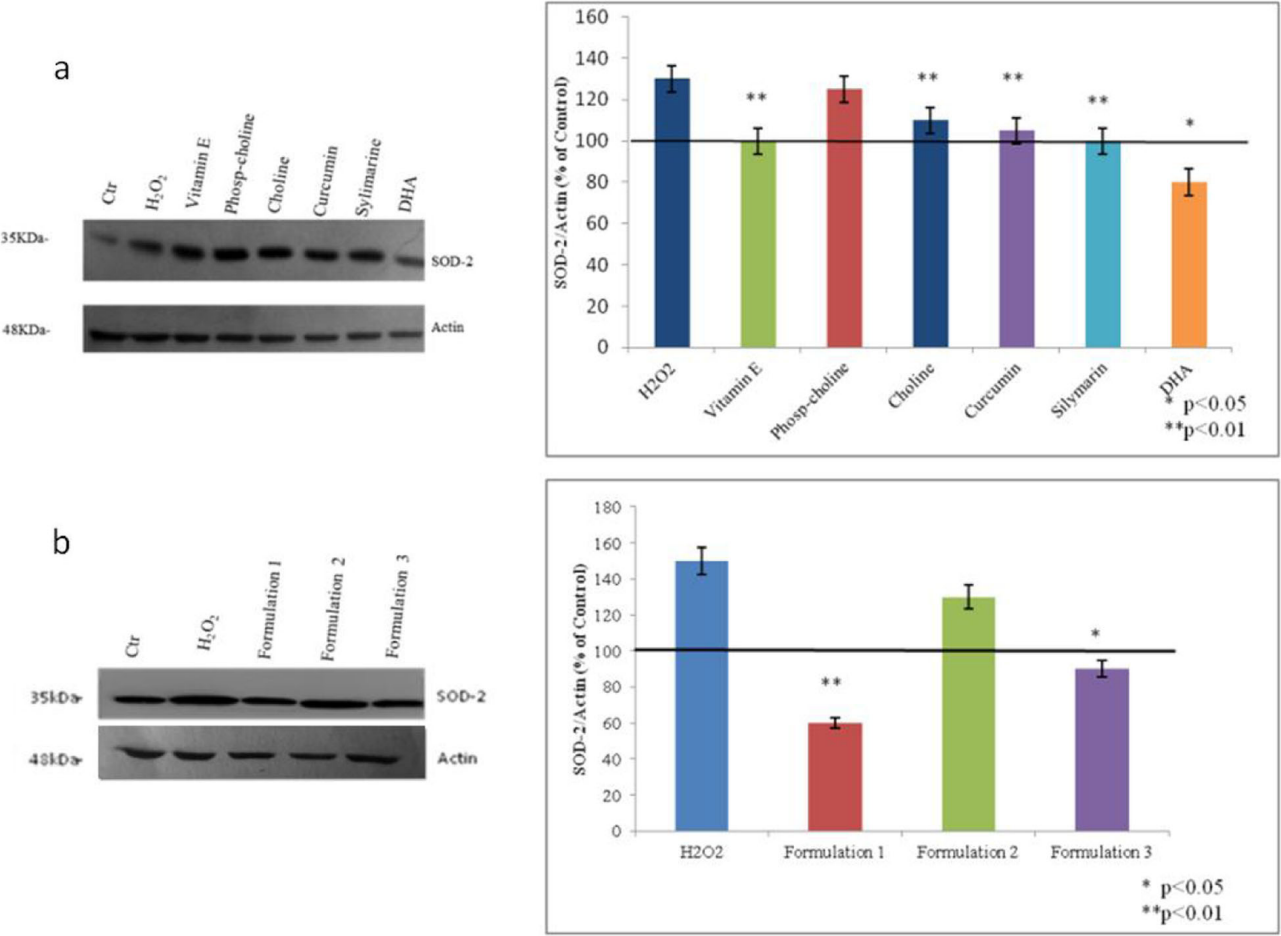
**a**, **b** Western blot relative to SOD-2 as a marker of oxidative stress. Actin is used to normalize the results. All values were expressed in the form of mean ± SD *(n = 3)*

**Fig. 6:**
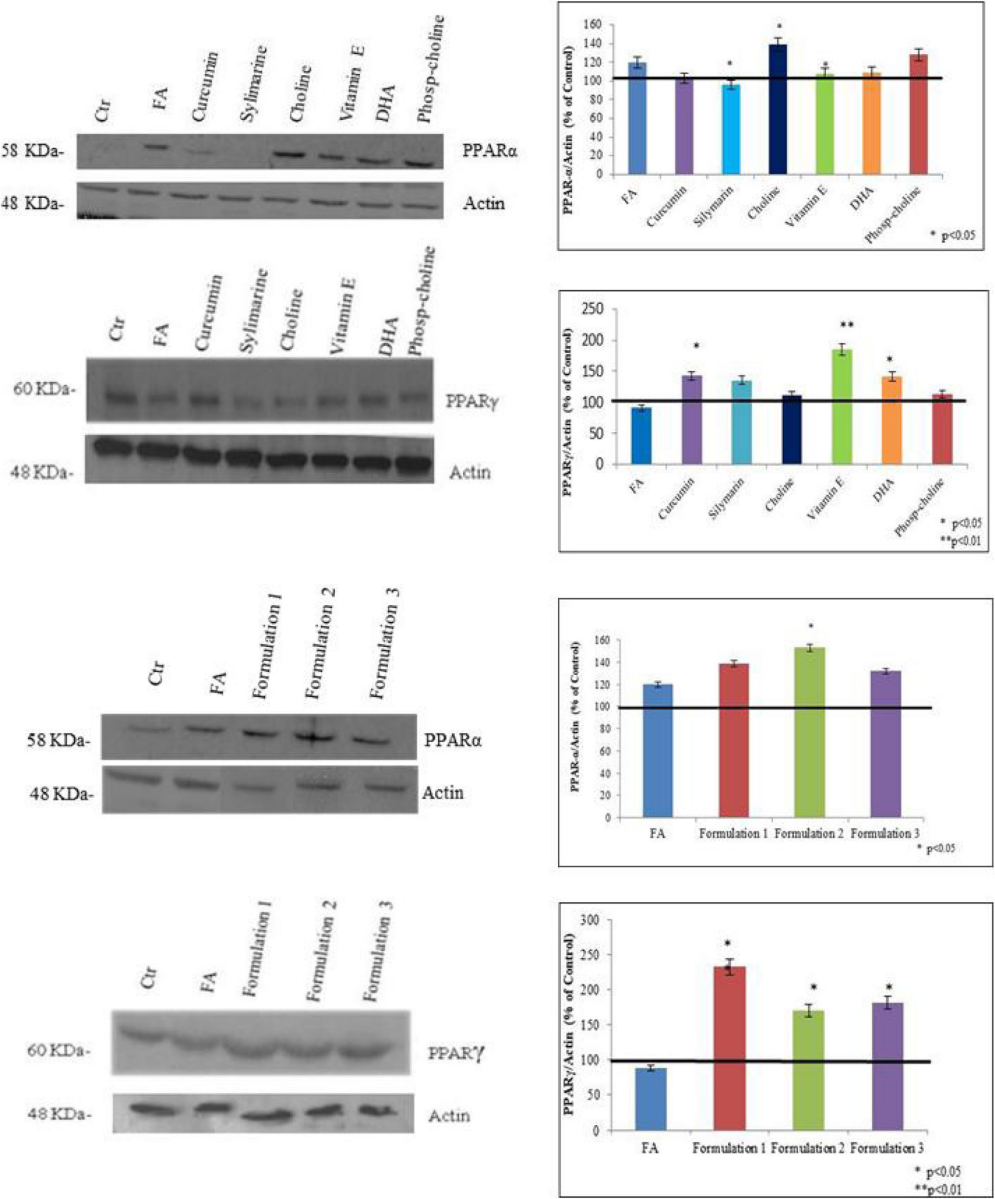
PPARα and PPARγ protein expression levels on HepG2 cells determined by Western blotting. Actin is used to normalize the results
